# MEPFeatX—automated feature extraction of motor-evoked potentials in transcranial magnetic stimulation

**DOI:** 10.3389/fnins.2024.1415257

**Published:** 2025-01-14

**Authors:** Dao T. A. Nguyen, Laura Säisänen, Elisa Kallioniemi, Pasi A. Karjalainen, Saara M. Rissanen, Petro Julkunen

**Affiliations:** ^1^Department of Technical Physics, University of Eastern Finland, Kuopio, Finland; ^2^Department of Clinical Neurophysiology, Kuopio University Hospital, Kuopio, Finland; ^3^Department of Biomedical Engineering, New Jersey Institute of Technology, Newark, NJ, United States

**Keywords:** feature extraction, motor evoked potential, automated analyses, transcranial magnetic stimulation, motor function

## Abstract

Motor evoked potentials (MEPs) are an important measure in transcranial magnetic stimulation (TMS) when assessing neuronal excitability in clinical diagnostics related to motor function, as well as in neuroscience research. However, manual feature extraction from large datasets can be time-consuming and prone to human error, and valuable features, such as MEP polyphasia and duration, are often neglected. Several packages have been developed to simplify the process; however, they are often tailored to specific studies or are not accessible. Here, we introduce MEPFeatX, a verified MATLAB package designed for automated and comprehensive MEP feature extraction across a wide range of stimulation paradigms. MEPFeatX is designed and documented for easy integration into any MEP analysis pipeline. Primed templates for specific paradigms, as well as additional analysis coded in R language, are also provided. Thus, MEPFeatX provides its users with a comprehensive and accurate set of MEP features, along with their visuals, facilitating quick and reliable MEP analysis in TMS studies.

## 1 Introduction

Motor evoked potentials (MEPs) are crucial in transcranial magnetic stimulation (TMS) studies. MEPs induced by TMS were first introduced by Barker et al. in 1985, when magnetic pulses were applied to a participant’s motor cortex and induced a muscle contraction in the participant’s hand. This demonstrated that MEPs can be used as a non-invasive assessment of corticospinal excitability (Barker et al., 1985; Rossini et al., 2015). Enabled by technological advancements in TMS, MEPs have been studied across various stimulation paradigms, either strengthening or challenging previous hypotheses and exploring novel mechanisms in many areas of neuroscience (Rossini et al., 2015; Siebner et al., 2022).

Each MEP feature provides unique insights into the underlying neurophysiological mechanisms (Rossini et al., 2015; Siebner et al., 2022). Peak-to-peak amplitude (Amp) and onset latency (Lat) are the two most commonly used MEP features. Amp represents the size of the MEP; a higher Amp indicates higher cortical excitation, as demonstrated by increasing stimulation intensity or navigating closer to the representative area of the targeted muscle at the primary motor cortex (Pellegrini et al., 2018). Lat and the time point of the first major peak (T1T) are both timing features of MEPs, but they reflect different neuronal mechanisms. Lat is the duration required for an activation potential to travel from the stimulated area to the distal muscle. Therefore, it has been utilized in studying central motor conduction mechanisms (Rusu et al., 2014). The T1T, or peak latency, is the time point at which Amp is the highest. It is almost consistent despite changes in *SI* and might reflect the precision of timed motor performance (Nguyen et al., 2023).

Less commonly studied features, such as MEP polyphasia and duration (Dur), offer valuable information about the corticospinal tract’s status. MEP polyphasia is characterized by the number of turns and phases (NT and NP, respectively) present in its waveforms. High MEP polyphasia indicates a hyper-excitable motor neuron system in multiple sclerosis (Perretti et al., 2004; Neva et al., 2016; Snow et al., 2019). Abnormal Dur has been detected in several movement disorders and motor neuron diseases, such as prolonged Dur in multiple sclerosis (Snow et al., 2019; Šoda et al., 2023) and shortened Dur in acute stroke (Brum et al., 2016). In addition to these intrinsic features, combined features can be derived from these original features, such as the area under the curve (AUC) and thickness, defined as the ratio of AUC to Amp (Sollmann et al., 2021).

**Figure 1:**
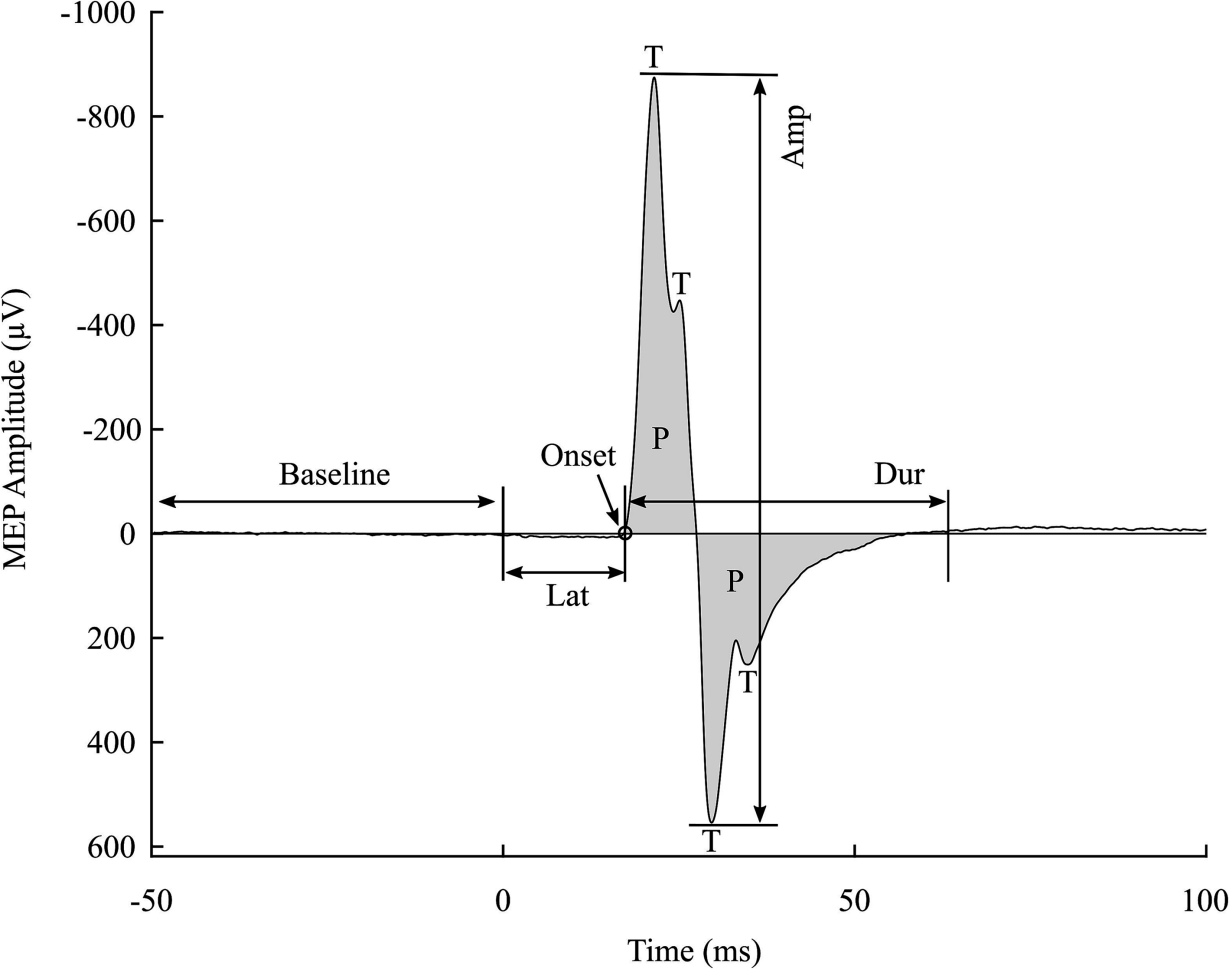
Visualization of an individual MEP with some of its features denoted. Amp, amplitude; Dur, duration; Lat, latency; P, phase; and T, turn. The two major peaks are denoted by T1 and T2, each providing a feature of timing and amplitude (T1T, T1A, T2T, and T2A). In addition to the number of turns (NT) and number of phases (NP), the area under the curve (AUC) is the sum of all phases’ area. Thickness is defined as a ratio of the AUC to Amp.

Despite the development of several toolboxes for MEP feature extraction, many are tailored to specific studies, such as recruitment curve analysis or online feature extraction, and often provide incomplete sets of MEP features. To address these limitations, this study introduces MEPFeatX, a robust package designed to automatically extract and visualize MEP features, covering essential information about MEP size, critical time points, and other morphological characteristics, including duration and polyphasia. Figure 1 illustrates an example of an MEP with its features extracted and visualized using MEPFeatX. This package was validated with MEPs recorded across various stimulation paradigms and is available at the following GitHub repository: https://github.com/NeuromodulationUEF/MEPFeatX. By clearly outlining the significance of MEP features and their applications, we aim to enhance users’ understanding of their practical utility in diverse contexts, thereby reinforcing the contribution of our work to the field of TMS research.

## 2 Existing tools for MEP feature extraction

The usual data pipeline in MEP studies includes data acquisition, preprocessing of raw electromyography (EMG) recordings, feature extraction from preprocessed MEPs, data analysis and visualization, and reporting. Data acquisition outputs the recorded EMG data and metadata of the experiment. Extracting the data from these formats requires a tailored process, depending on the devices/software used. Preprocessing includes filtering, artifact removal, and segmentation of data into trials. Next, features of interest are extracted from each trial, analyzed using appropriate statistical methods, and visualized to facilitate the interpretation of the results. Reports are generated afterward.

Several toolboxes have been developed over the years for various steps in this data pipeline, such as MAVIN, CortExTool, Motometrics, VETA, and MEP-ART. A summary of these toolboxes is provided in Table 1.

**Table 1:** Existing toolboxes for MEP feature extraction.

Toolbox	Features	Additional functions	Tested data	Tested paradigms
MAVIN	Amp Lat	MEP visualization: EMG long train and MEP segments	41 healthy controls and substance-dependent individuals Hand muscle	Single-pulse TMS Paired-pulse TMS: interhemispheric inhibition/facilitation CSP measurement Recruitment curve fitting
CortExTools	Amp Lat Dur Mean RMS	Signal preprocessing, artifact detection, MEP detection CSP detection Recruitment curve fitting Motor mapping recreation	Healthy controls Hand muscle	Baseline Paired-pulse TMS CSP measurement Motor mapping Simulated data
Motometrics	Amp AUC RMS Lat	Datafile annotation Near real-time feedback during experimental procedures Signal preprocessing Recruitment curve analysis	No information	No information
VETA	Amp Lat AUC Dur	Online data collection and visualization Signal preprocessing, MEP detection CSP detection	Four healthy participants Hand muscle	Paired-pulse TMS: SICI Stop task Delayed response task CSP measurement
MEP-ART	Amp	Real-time feedback during measurement Recruitment curve fitting	No information	Recruitment curve fitting Paired-pulse TMS: SICI, ICF

Amp, Amplitude; AUC, area under the curve; CSP, cortical silent period; Dur, duration; EMG, electromyograph; ICF, intracortical facilitation; Lat, Latency; MEP, motor evoked potential; RMS, root mean squared; SICI, short interval intracortical inhibition; TMS, transcranial magnetic stimulation.

*MAVIN* (Mullins and Hanlon, 2016) is an open-source tool for offline MEP visualization and analysis and can be utilized in studies involving basic/paired associative stimulation, as well as the cortical silent period (CSP) and recruitment curves. *Motometrics* (Ratnadurai Giridharan et al., 2019) focuses specifically on recruitment curve analysis.

*VETA* (Jackson and Greenhouse, 2019) provides real-time data acquisition and feature extraction. *MEP-ART* (Skelly et al., 2020), is a real-time feedback and MEP analysis tool used to investigate the reliability of recorded data. It is compatible with the Magstim 200 (and BiStim) system (Magstim Inc., Eden PrairieMN). *VETA*, on the other hand, focuses on detecting MEPs from EMG recordings and then analyzes and visualizes the detected MEPs. *CortExTool* (Harquel et al., 2016) is a comprehensive MATLAB toolbox designed for MEP analysis. It includes features for EMG preprocessing, automatic MEP detection, the extraction of various MEP features, and the analysis of I/O curves and cortical silent periods.

MAVIN and Motometrics were inaccessible. In addition, we were unable to run *MEP-ART*, *CortExTool*, and *VETA*, primarily due to incompatible data formats.

## 3 Methods

### 3.1 Design

In comparison to the existing toolboxes mentioned in Section II, MEPFeatX focuses on feature extraction. In total, it outputs a set of 11 MEP features: Amp, Lat, T1T, Dur, AUC, NT, NP, amplitude of the first major turn (T1A), time point of the second major turn (T2T), amplitude of the second major turn (T2A), and thickness.

The first version of the package was developed to explore MEP patterns in single-pulse TMS performed at the cortical representation area for the hand and to estimate the minimum number of trials required to reliably represent the entire dataset using bootstrapped principal component regression (Nguyen et al., 2019). The current version of this package was further improved to offer greater flexibility, allowing it to work on MEPs in various conditions, such as across multiple age groups, muscles, and stimulation paradigms (Table 2) (Nguyen et al., 2019, 2023). The use cases can be found on the MEPFeatX Wiki, under the “Use cases” page.

**Table 2:** Metadata table for the sample datasets.

Sample	Paradigm	Protocol	Target muscle	Experimental design
1	Single-pulse TMS	Single pulse	Right FDI	152 single pulses at 120% rMT
2	Long-interval cortical inhibition	Paired pulse	Right TA	20 bursts, each containing two pulses at 120% rMT at a frequency of 0.1 Hz.
3	Repetition suppression	rTMS	Right TA	20 bursts, each containing four pulses at 120% rMT at a frequency of 1 Hz.
4,5,6	Short-interval intracortical facilitation	Paired pulse	Right TA	20 bursts, each containing two pulses at 120% rMT. Three sequences were performed with inter-trial intervals of 1.4, 3.0, and 7.0 ms, respectively.
7	Coil orientation	Single pulse	Right FDI	120 single pulses at 120% rMT with the variation in the coil angle
8	Recruitment curve	Single pulse	Right ECR	70 single pulses at 90–150% rMT
9	Recruitment curve	Single pulse	Left ECR	70 single pulses at 90–150% rMT

rMT, resting motor threshold; TA, tibialis anterior; FDI, first dorsal interosseous; ECR, Extensor carpi radialis; rTMS, repetitive TMS.

**Figure 2:**
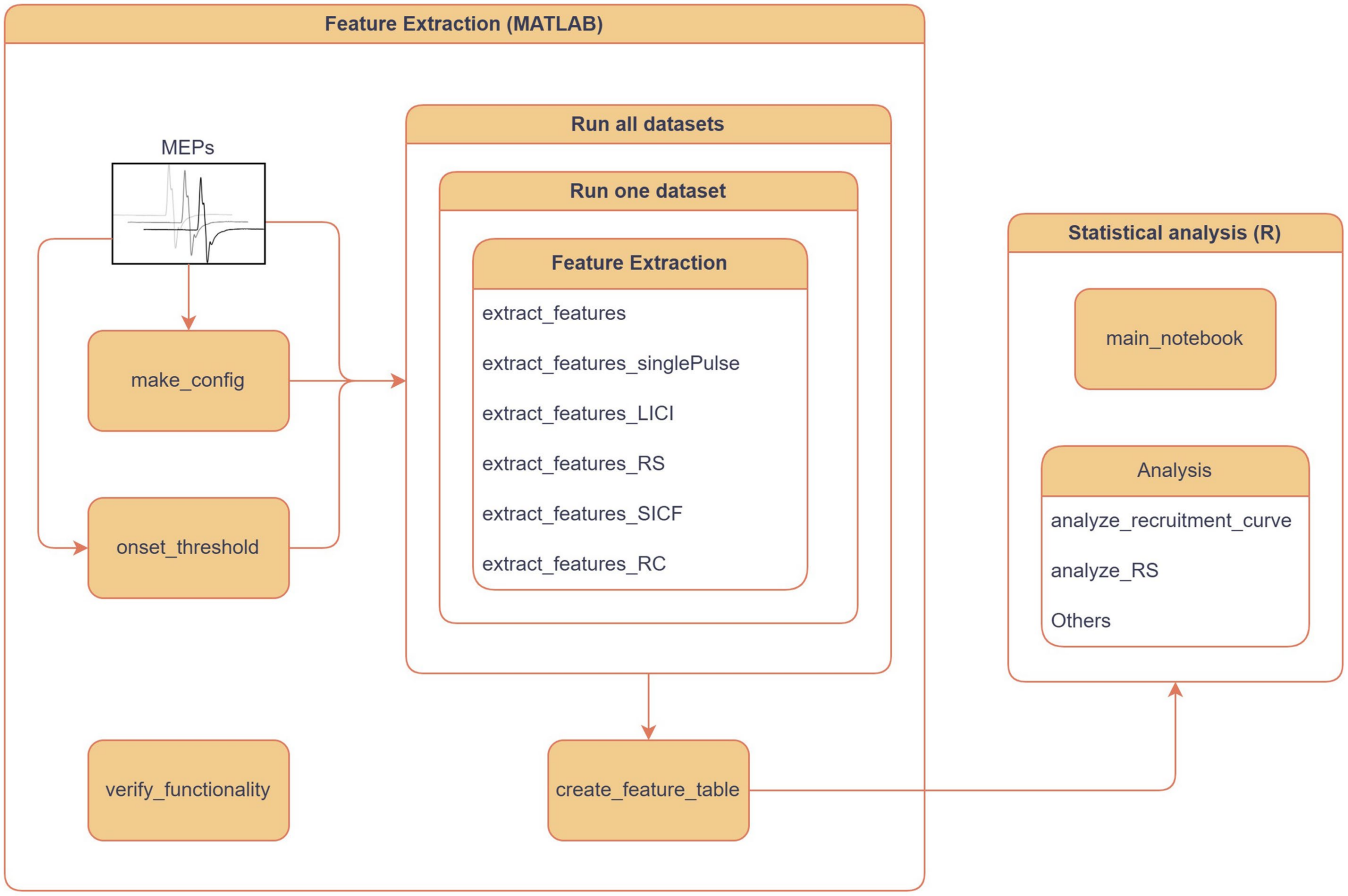
MEPFeatX workflow. The primary component of MEPFeatX is feature extraction, programmed in MATLAB, designed to extract features from MEP responses across various stimulation paradigms. Users can select from several paradigms, including long-interval intracortical inhibition (LICI), repetition suppression (RS) at 1 Hz, and short-interval intracortical facilitation (SICF). Once the feature table is generated in MATLAB, users can utilize R notebooks for further analysis, including descriptive statistics and linear mixed-effects modeling.

In addition to feature extraction, the package also provides a workflow for feature extraction, particularly for MEPs, including a combination of metadata and features as a comprehensive table that can be utilized for exploratory data analysis, logs of the extraction process, and reliable evaluation of feature quality through plots. Documentation on MEPFeatX use cases was created to enhance the package’s usability and ensure the reproducibility of its functions in any MEP analysis pipeline (Romano and Moore, 2020). Furthermore, the user can select several stimulation paradigms, such as long-interval intracortical inhibition (LICI), repetition suppression (RS) at 1 Hz, and short-interval intracortical facilitation (SICF). After the feature table is created in MATLAB, the user can opt to use R notebooks for analyzing the table. Several R scripts are provided to analyze the exported feature table through descriptive analysis and/or causal analysis to identify the decisive factors in each study, such as stimulation protocols and subject demographics (Figure 2).

MEPFeatX offers a more streamlined approach focused solely on feature extraction compared to the existing toolboxes. It accepts MATLAB .mat files as input, eliminating data format mismatches. In addition, MEPFeatX is fully and freely available to the community, with version control for both code and documentation managed through Git. Updates and bug fixes for the package can be requested via GitHub or directly from the authors. Version control is implemented for the package scripts and analysis. MEPFeatX outputs are automatically organized into specified categories and saved to folders named by the date the analysis is performed.

MEPFeatX does not include a graphical user interface (GUI) as a GUI requires a specific version of the MATLAB runtime. Instead, the package workflow is programmed similarly to the R Markdown Notebook (Allaire et al., 2024) and Python Jupyter Notebook (Kluyver et al., 2016), with ample comments for easy integration into any analysis pipeline.

### 3.2 Package components

Given that MEPs are already segmented from the recorded EMG, the core functionality of MEPFeatX is the feature extraction algorithm, which simply follows the definition of each feature. First, it detects the two most prominent peaks (positive and negative) appearing in a signal segment from the TMS pulse delivery time to 150 ms afterward. Amp, T1T, T1A, and T2T are extracted from the timing and magnitude of these two peaks.

We used the standard deviation of the 50 ms segment prior to the delivery of the TMS pulse as the background activity. The MEP onset is the time point when the signal first exceeds the background activity within the time window from the pulse delivery time to the T1T. Lat is the time interval between the pulse delivery time and the MEP onset. The endpoint of the MEP is defined as the point when the signal returns to the level of the background activity. It is determined as the first sample of a 10 ms signal segment that is statistically similar to the background activity, meaning this 10 ms segment has a standard deviation comparable to that of the background activity. Dur is the time interval between the MEP onset and its endpoint. Next, the NT is counted as the significant peaks occurring during the MEP *Dur*, while the NP is counted by the zero-crossing points between the MEP onset and endpoint. Combined features, such as AUC and thickness, are calculated afterward. The AUC is the area under the rectified signal during the Dur. Thickness is the ratio of the AUC to Amp. The visualization of MEPs and their features is shown in Figure 1.

### 3.3 Prerequisites

For the current version, the package runs on preprocessed MEP datasets stored in MAT files, and the input dataset must have rows as samples and columns as a stack of trials.

A ready-made configuration file contains control parameters for running MEPFeatX, such as specifying data directories, creating new folders for storing outputs, and defining the sampling frequency. All these parameters must be checked carefully for the extraction to run. Details on the data format and configuration file are provided in the MEPFeatX GitHub Wiki.

### 3.4 Workflow

A complete workflow is provided with the package to help the user create a new analysis (Figure 2). This workflow demonstrates feature extraction on all datasets or a single dataset stored in the data folder and saves the output to the analysis folder. Therefore, the user can access the data at different stages of the analysis, such as figures, features, or statistical analysis results.

Logs of the current analysis are also recorded and saved to the analysis folder. Errors, such as missed MEPs where the extraction failed, are reported in a table-format file for review. Details on the deployment of MEPFeatX are provided in the MEPFeatX GitHub Wiki.

## 4 Results

### 4.1 Package verification and validation

MEPFeatX functions were utilized in our previous studies (Nguyen et al., 2019, 2023). The two studies used single-pulse TMS in healthy participants across four age groups: children, preadolescents, adolescents, and adults. MEPs were recorded from upper extremity muscles, such as those in the forearm and hand.

MEPFeatX was validated using a comprehensive dataset of participants from several studies on MEP analysis, two of which have been published (Nguyen et al., 2019, 2023). In the 2019 study (Nguyen et al., 2019), the studied dataset contained data samples from nine healthy adult participants. Each sample included 120 MEPs at a stimulation intensity of 120% rMT. In the 2023 study (Nguyen et al., 2023), 38 healthy participants were categorized into four age groups: children, preadolescents, adolescents, and adults. A total of 70 stimuli were performed with the stimulation intensity (SI) randomly ranging from sub-threshold to supra-threshold. MEPs were recorded from the flexor carpi radialis (FCR), extensor carpi radialis (ECR), abductor digiti minimi (ADM), and first dorsal interosseous (FDI) muscles. Stimulation was performed on both hemispheres, and EMG was recorded from the contralateral muscles. The MEPFeatX output was manually verified in these studies. Thus, this demonstrated the capabilities of MEPFeatX in extracting MEP features across various age groups, muscle groups, and stimulation protocols.

To verify the output of MEPFeatX in lower extremity muscles and other paradigms, we extracted MEP features from 7,799 MEPs recorded from the tibialis anterior (TA) muscle across six paradigms: single-pulse TMS, repetition suppression (RS) at 1 Hz, long-interval intracortical inhibition (LICI) at 100 ms, and short-interval intracortical facilitation (SICF) with three inter-pulse intervals of 1.4 ms, 3.0 ms, and 7.0 ms. Data were collected from 23 healthy controls. The studies involving human participants were reviewed and approved by the Research Ethics Committee of the Hospital District of Northern Savo. The participants provided written informed consent to participate in this study.

**Figure 3:**
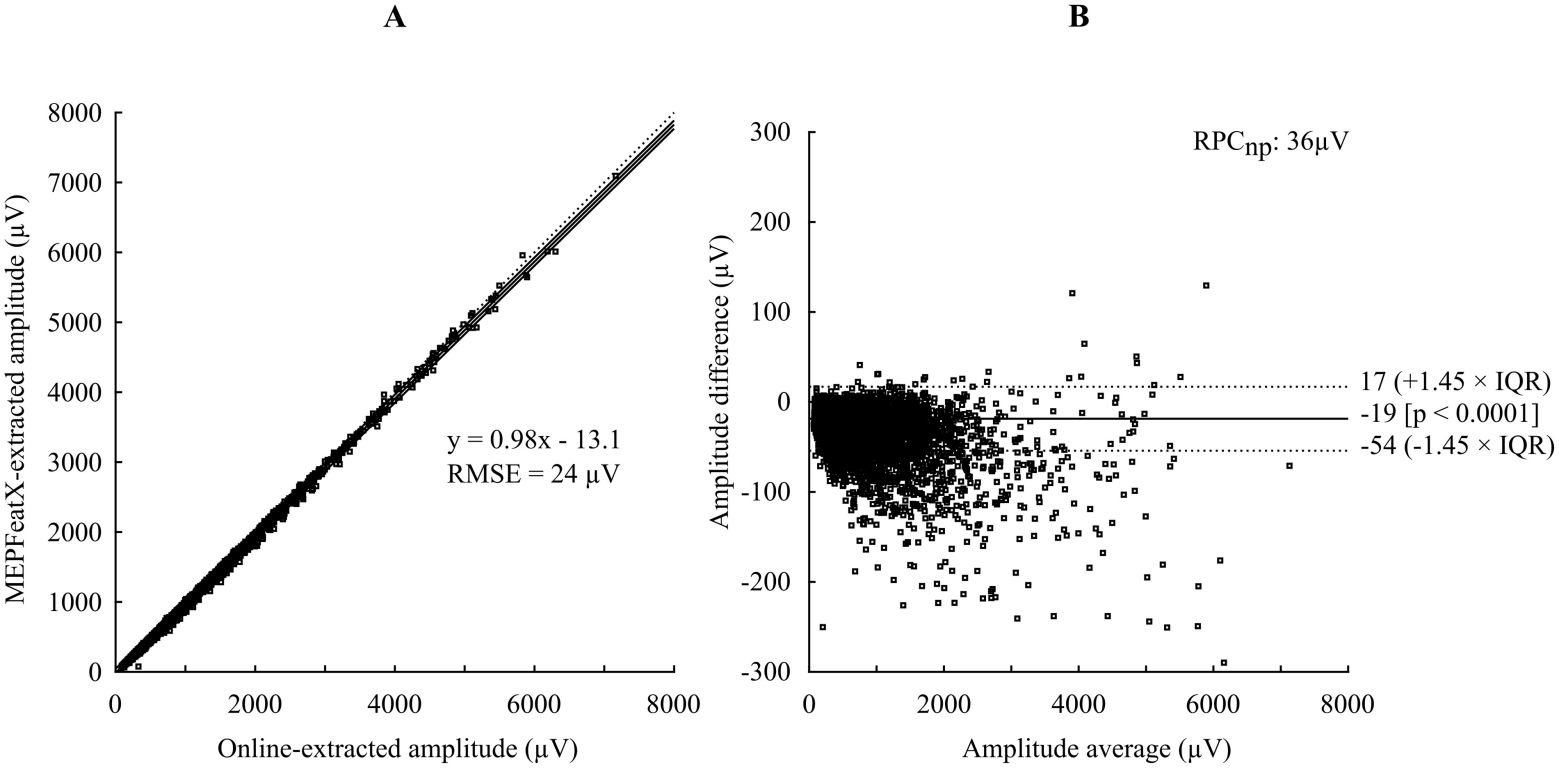
A comparison of online-extracted amplitude and MEPFeatX-extracted amplitude using Bland–Altmann analysis. **(A)** A regression plot between the amplitude extracted by the online algorithm and MEPFeatX. **(B)** A plot of pair-wise differences of the amplitude extracted by the two methods. The amplitude extracted by MEPFeatX was lower than the online-extracted amplitude by 19 μV, and the bias became larger with higher amplitude. This bias was due to the effect of the low-passed filter during the preprocessing in MEPFeatX, which reduced high-frequency noises. RMSE, root mean squared error; IQR, inter-quartile range; RPCnp, non-parametric coefficient of reproducibility.

Bland–Altman analysis was used to compare the Amp extracted by MEPFeatX with the Amp extracted in real-time during the data acquisition (Figure 3). The Pearson correlation coefficient between the Amp extracted by the two algorithms was *r* = 0.998 (*p* < 0.0001), with a coefficient of reproducibility of 36 μV. The online-extracted latency was unreliable and was not compared.

It is recommended to validate the package’s functionality before its first use in a new system. The package includes outputs from anonymized sample datasets and the validation function. The features extracted from the sample datasets and their plots are used as a reference to validate the package’s output before the first use.

### 4.2 Sample datasets and analysis templates

The sample datasets and their analysis templates are provided to help users implement MEPFeatX in their own studies (Table 2). Each sample dataset contains raw responses and preprocessed MEPs, both of which have been segmented into 200 ms segments, from 50 ms prior to TMS administration to 150 ms after the pulse. The provided templates help users manipulate and visualize MEP features in specific paradigms. For a detailed explanation, refer to the MEPFeatX GitHub Wiki.

## 5 Discussion

This study aimed to enhance the analysis of MEPs by introducing the MEPFeatX package. Our findings indicate that MEPFeatX significantly improves the automatic and comprehensive feature extraction of MEPs across various stimulation paradigms.

When comparing MEPFeatX to existing tools, we found that MAVIN and Motometrics were not accessible for our analysis. In addition, MEP-ART, CortExTool, and VETA could not be executed on our data due to input format mismatches. In contrast, MEPFeatX not only provides a user-friendly interface but also includes visualizations that reliably evaluate analyzed MEPs and their features. Furthermore, the package offers workflows and templates for several use cases, which enhances reproducibility and integrity within MEP analysis pipelines. In addition, MEPFeatX is designed to efficiently handle datasets of varying sizes. The MATLAB codes are segmented into small functions that support parallel computing. This feature allows it to effectively run large datasets.

Despite these advantages, our study has limitations. The accessibility issues with other tools highlight the need for data pre-processing. Thus, it might not work with EMG signals that contain significant interference. Future research should focus on exploring the application of MEPFeatX across additional paradigms to further validate its effectiveness in diverse settings.

Although the output of MEPFeatX was visually observed by two experts in the field, we did not record any objective metrics. Thus, future development and validation of MEPFeatX should follow a structured procedure that includes human evaluation with proven metrics, such as the level of agreement among evaluators.

In conclusion, MEPFeatX emerges as a superior tool for MEP analysis, allowing researchers to focus on exploratory analysis and derive meaningful insights from exported features. We encourage further investigations to expand its applicability and address existing limitations.

## Data Availability

The data analyzed in this study is subject to the following licenses/restrictions: the data that support the findings of this study are available from the corresponding author, DN, upon reasonable request. Requests to access these datasets should be directed to Dao T. A. Nguyen, nguyen.dao.bk@gmail.com.
